# Evaluation of Distortion and Transient Evoked Otoacoustic Emission in Tinnitus Patients with Normal Hearing

**Published:** 2014-01

**Authors:** Helnaz Mokrian, Abdolreza Shaibanizadeh, Saeid Farahani, Shohreh Jalaie, Parvane Mahdi, Amin Amali, Homa Arian nahad

**Affiliations:** 1 *Department of Audiology, Faculty of Rehabilitation Sciences, Iran University of Medical Sciences, Tehran, Iran.*; 2 *Department of Audiology, Faculty of Rehabilitation Sciences, Tehran University of Medical Sciences, Tehran, Iran.*; 3 *Department of Statistics, Faculty of Rehabilitation Sciences, Tehran University of Medical Sciences, Tehran, Iran.*; 4 *Department of Otorhinolaryngology-Head and Neck Surgery, Imam Khomeini Educational Complex Hospital, Tehran University of Medical Sciences, Tehran, Iran.*

**Keywords:** Distortion-Product Evoked Otoacoustic Emissions, Normal hearing, Subjective Tinnitus, Transient Evoked Otoacoustic Emission

## Abstract

**Introduction:**

Tinnitus is a perception of sound without external source. The exact etiology of tinnitus is not fully understood, although some researchers believe that the condition usually starts in the cochlea. The aim of this study was to determine the potential contribution of outer hair cell dysfunction to chronic tinnitus, by application of Distortion-Product Evoked Otoacoustic Emission (DPOAE) and Transient Evoked Otoacoustic Emission (TEOAE) and also to determine the relationship between tinnitus loudness and the amplitude of these two potentials.

**Materials and Methods:**

This study was conducted on 20 tinnitus patients aged 20–45 years and 20 age- and gender-matched control subjects. DPOAE and TEOAE were performed on each subject.

**Results:**

The difference in the amplitudes of TEOAE between the two groups was not significantly different (P=0.08), but the amplitude of DPOAE in patients with tinnitus was significantly lower than the corresponding value in the control subjects (P=0.01). There was no correlation between tinnitus loudness and the amplitudes of neither DPOAE nor TEOAE.

**Conclusion:**

Abnormal findings in the DPOAE of tinnitus sufferers suggest some form of cochlear dysfunction in these patients. As there was no correlation between the amplitude of the recorded potentials and tinnitus loudness, factors other than cochlear dysfunction may also influence the loudness of tinnitus.

## Introduction

Tinnitus, from the Latin word tinnire, meaning “to ring,” is a phantom perception of sound when no such sound is present externally (1). Tinnitus affects the quality of life of millions of people around the world and is associated in most cases with hearing impairment (2). Although most incidences of tinnitus are temporary, chronic subjective tinnitus occurs in 4–15% of the general population, with the prevalence increasing in those above 50 years of age to almost 20% (3). Broadly speaking, approximately 90% of individuals with chronic tinnitus have some form of hearing loss; however, only about 30–40% of those with hearing loss develop tinnitus (4,5). For instance, Barnea et al. (1990) found that 8% of patients with tinnitus had normal hearing thresholds up to 8,000 Hz (defined as better than or equal to 20 dBHL) (6). As reported by the National Center for Health Statistics, tinnitus affects approximately 12% of people between the ages of 65–74 years, and the prevalence of hearing impairment in the tinnitus population is between 80 and 90% (1).

Many theories have been presented in an attempt to explain the origin of tinnitus in patients with normal hearing. Although tinnitus may be associated with abnormalities at any level of the auditory pathway, it very often starts in the cochlea. Indeed, Jastreboff et al (1993) considers that tinnitus usually starts in the cochlea and generates abnormal activity in the central pathways, which prolongs the systems; the central auditory pathways do not need to be structurally altered (7). It was hypothesized that small cochlear abnormalities can result in tinnitus, even before any hearing loss is apparent (8,9).

Otoacoustic emissions (OAEs) are a propagation of the audiofrequency signal into the ear canal from the cochlea, transmitted through the ossicular chain and tympanum. They are believed to originate from an active mechanical force generated by the outer hair cells (8).

OAEs are an important tool for the objective evaluation of the inner ear function, especially the outer hair cells of the cochlea. They occur in response to acoustical stimulation, or can even appear spontaneously. Otoacoustic emissions can be evoked by a broadband stimulus (a click) or by brief tones (9). 

As otoacoustic emissions are invariably associated with functioning outer hair cells (OHC), their presence is a reliable indicator of cochlear structural integrity, and their absence may indicate a cochlear lesion. Furthermore, by recording otoacoustic emissions, a subclinical cochlear lesion may be detected, as up to 30% of the OHC population may experience damage before any audiometric evidence is apparent in the quarter octaves pure-tone audiometry from 0.125 kHz to 16 kHz. Therefore, we selected this test to evaluate the cochlea in tinnitus patients (10). 

The aim of this study was to evaluate cochlear function in tinnitus patients; in other words, we wanted to determine whether or not tinnitus occurs because of a dysfunction at the level of the OHCs. In this study, we used evoked otoacoustic emissions (EOAE) to assess the function of the OHCs in tinnitus patients with normal hearing.

## Materials and Methods

*Subjects: *The patient group consisted of 20 adult males with subjective tinnitus (bilateral or unilateral) who had normal hearing thresholds as revealed by pure-tone and speech audiometry measurements who attended an audiology clinic in a university center, from June 2011–July 2012. Normal hearing was defined as a hearing level of 20 dB or less at all audiometric frequencies (250, 500, 1,000, 2,000, 4,000, 6,000, 8000, 12,000, and 16,000 Hz). 

 A group of 20 healthy male subjects with normal hearing and no history of tinnitus or auditory or vestibular symptoms participated as a control group. The two populations had similar gender and age distribution. All patients had a definitive diagnosis of subjective tinnitus that lasted more than 1 month, as well as a thorough medical history and clinical examination verified by an otologist.

 The following exclusion criteria for both the patient and control groups were applied at baseline: history of familial hearing loss, tinnitus perception less than 1 month, use of ototoxic drugs, chronic otitis media, head trauma, and age >45 years.

*Audiometric Test Batteries*

All subjects underwent the following test battery: pure-tone audiometry, tympanometry (frequency, 226 Hz) and stapedial muscle reflex, tinnitus pitch-matching procedures, tinnitus loudness-matching procedures, Transient Evoked Otoacoustic Emission (TEOAEs), and Distortion-Product Evoked Otoacoustic Emission (DPOAEs).

Subjects were seated in an acoustically test chamber. First, pure-tone audiometry, tympanometry, and stapedial muscle reflex were performed, and then the pitch and loudness of tinnitus was evaluated. In this measurement we asked the patient to compare the pitch produced by a noise or pure tone to the most prominent pitch of tinnitus. They then adjusted the intensity of a noise or tone so that it had the same loudness as their tinnitus. 

Next we evaluated otoacoustic emission. The first ear examined was the right ear in patients with bilateral tinnitus; while in unilateral patients we first assessed the affected ear. TEOAE testing always was performed first. TEOAEs were performed with a wide band click in a continuous mode and an intensity of 80 dB peak sound pressure level (SPL). For measuring distortion product (DP) grams, the frequency separation of the primaries was f2/f1 = 1.22, with L1 and L2 set to 65 and 55 dB SPL, respectively.

The following parameters were considered in the TEOAE tests: 1) signal-to-noise (S/N) ratio_6 dB 2) and reproducibility of the responses of at least 70%. In the DPOAE, the parameters of interest in all tested frequencies were: 1) S/N ratio_6 dB in; 2) amplitude of the signal in the 90^th^ percentile of the normal distribution for the frequencies tested; 3) only the frequencies from 1,000 to 8,000 Hz were considered.

*Statistical Analysis*

All data were analyzed using SPSS version 16.0 (Chicago, IL, USA), including routine statistical procedures such as Leven’s independent *t *test, Mann-Whitney *U* test, and one-way analysis of variance (ANOVA), at the significance level of P<0.05.

## Results

Mean (± standard deviation) of age was 29/10 (± 6/43) years for patients and 28/40 (±6/02) years for controls. There was no statistically significant difference between the two groups with respect to gender or age (P>0.05).

*Pure-Tone Audiometry*

Results of pure-tone audiometry confirmed normal hearing in all subjects, and there were no significant differences in the mean threshold levels between the groups (P>0.05). The results of audiometric test are shown in (Table. 1).

**Table 1:** Mean (standard deviation) pure-tone audiometry results

	Threshold/ Left	Threshold/ Right	
Control Subjects	8/35(4/18)	7.40 (3.99)	
Patients Subjects	9/08(3/51)	7.55 (3.67)
P- Value	0.103	0.317

*Tympanometry*

Tympanometric measures in all subjects were within normal ranges and the middle ear pressure was comparable between the groups.

*Acoustic Reflexes*

In all subjects, acoustic reflexes, ipsilateral and contralateral, were lower than 110 dB across at least three adjacent frequencies.

*Otoacoustic Emission Tests*

We chose TEOAE and DPOAE because they are the most commonly used OAE tests in clinical practice and also they have more standardized methodology. The criteria for normality are stated in the Materials and Methods section. 

*TEOAEs*

There was no significant difference between the mean of overall TEOAE amplitude in patients with bilateral tinnitus, unilateral tinnitus, and normal subjects (P>0.05) (Table.2).

**Table 2:** Mean (standard deviation) TEOAE amplitude (dB SPL).

	Amplitude/ Left	Amplitude/ Right
Control Subjects	12.74 (4.21)	12.55 (3.71)
Patients Subjects	12.77 (5.41)	13.77 (5.07)
P- Value	0.703	0.512

*DPOAEs*

The mean of overall DPOAE amplitudes in patients with tinnitus was significantly lower than the corresponding value in normal subjects (P<0.05); however, there was no difference between patients with unilateral and bilateral tinnitus (Table. 3).

**Table 3:** Mean (standard deviation) DPOAE amplitude (dB SPL)

	Amplitude/ Left	Amplitude/ Right
Control Subjects	9.23 (3.08)	9.97 (2.01)
Patients Subjects	6.66 (2.45)	7. 46 (3.27)
P- Value	0.006*	0.008*

* statistically significant

Our comparison between the amplitude of DPOAE in tinnitus patients and normal subjects was in the frequency of tinnitus, which we defined through the pitch-matching test for each patient.

*Correlation Between Tinnitus Loudness and DPOAE Results*

Pearson’s correlation coefficient was used to evaluate the degree of agreement between tinnitus loudness and DPOAE in the tinnitus patients. There was no correlation between tinnitus loudness and the amplitude of DPOAE in patients with tinnitus (P>0.05).

## Discussion

Tinnitus is an otologic symptom and, despite considerable research in this field, the exact pathophysiological processes of tinnitus remain unknown. Involvement of the whole auditory system, either peripheral or central, should be considered in the generation of tinnitus.

Some hypothesized pathomechanisms and causes of tinnitus include spontaneous otoacoustic emissions, edge theory, discordant theory, dorsal cochlear nucleus, hyperactivity/plastic readjustment of dorsal cochlear nuclei (DCN), crosstalk theory, pharmacological causes, and neurologic disorders(11).As the cause and the perception of tinnitus are so heterogeneous, an etiologic subdivision of tinnitus population is essential to achieve an effective treatment (12). 

The high incidence rate of cochlear damage and studies of cochlear mechanics have led to the suggestion that tinnitus at themed and high frequencies might, in many cases, arise from the cochlea; although the possibility that an acute exacerbation of tinnitus may result from changes in the efferent nervous system has also been suggested (13). A cochlear disorder, even when undiagnosed by pure-tone audiometry, may initiate a series of processes in the nervous system that may result in tinnitus (15).

Our objective was to verify cochlear dysfunction in patients with normal hearing who have tinnitus with the use of the TEOAE and DPOAE tests, and to evaluate the correlation between tinnitus loudness and amplitudes of the recorded potentials. 

In our study, there was no difference in TEOAE amplitude between tinnitus patients and normal subjects. This is consistent with a study by Geven et al (2011) who selected patients from a general tinnitus population and found no difference in TEOAE parameters between patients with tinnitus and normal subjects (14). However, this was in contrast with a study by Ceranic et al (1998) that was performed in tinnitus patients with head trauma, in which higher TEOAE amplitudes were reported in subjects with tinnitus compared with those without tinnitus (15). Szutka et al (2010) reported markedly higher DPOAE amplitudes in tinnitus patients without hearing loss and they explained that tinnitus may be caused by increased motility of the OHCs induced by decreasing efferent fiber activity, and not by OHC failure (16). The results of a study by Gouveris H et al (2005) were more complicated, as they reported that ears in which tinnitus is present exhibited relatively increased amplitudes of DPOAE at high frequencies (4–6.3 kHz) when compared with normal controls, and relatively decreased DPOAE amplitudes at mid frequencies (1650–2400 Hz) (17). 

In addition, Granjeiro et al (2008) reported that patients with tinnitus had a significantly higher prevalence of abnormal TEOAEs and DPOAEs than normal subjects (7). Fernandes et al (2009) found that the amplitude of TEOAE was lower in groups with tinnitus (18).

In our study, patients with tinnitus showed significantly lower DPOAE amplitudes than subjects who had no auditory complaints, suggesting OHC dysfunction. This observation is in agreement with studies by Ami et al (2008) and Hesse et al (2005), of DPOAE in tinnitus sufferers which showed that reduced OHC activity, as detected by reduced DPOAE levels, may manifest as tinnitus even before there is a shift in hearing threshold (19,20). 

We assumed that the difference between the results of TEOAE and DPOAE is because the DPOAEs are considered to have a higher sensitivity. Therefore, we recorded DPOAE with a lower amplitude in comparison with TEOAE. We found no relationship between tinnitus loudness and the magnitude of OHC damage, meaning that factors other than cochlear dysfunction may affect tinnitus loudness.

## Conclusion

Patients with normal hearing who have tinnitus may have cochlear damage. Although referring to a limited population, this observation might be useful in the attempt to elucidate the mechanisms of tinnitus generation and perception. We concluded that DPOAE is a more useful tool in an estimating cochlear damage in tinnitus patients with normal hearing. Further studies with a larger sample size and using other audiometric tests are needed for further evaluation of the mechanism of tinnitus generation.

## References

[1] Fatima T, Husain REM, CarolineWD, Yvonne SB, KristinaS, Nathan MP (2011). Neuroanatomical changes due to hearing loss and chronic tinnitus: A combined VBM and DTI study. brain research.

[2] Larry E. Roberts, Jos J.Egermont, Donald M. Caspary, Susan E. Shore, Jennifer R. Melcher, James A. Kaltenbach (2010). Ringing Ears: The Neuroscience of Tinnitus. J Neurosci.

[3] Moller AR (2007). Tinnitus: presence and future. Brain Res.

[4] Davis A, Rafaie EA, Tyler RS (Tinnitus Handbook). Epidemiology of tinnitus. Tinnitus Handbook.

[5] Lockwood AH, Salvi RJ, Coad ML, TowsleyML, Wack DS, Murphy BW (1998). The functional neuroanatomy of tinnitus:evidence for limbic system links and neural plasticity. Neurology.

[6] Barnea G, Attias J, Gold S, Shahar A (1990). Tinnitus with normal hearing sensitivity: extended high-frequency audiometry and auditory-nerve brain-stem-evoked responses. Audiology.

[7] Granjeiro RC, Kehrle HM, Bezerra RL, Almeida VF, Sampaio ALL, Oliveira CA (2008). Transient and distortion product evoked oto-acoustic emissions in normal hearing patients with and without tinnitus. Otolaryngology-Head and Neck Surgery.

[8] WE B (1990). Outer hair cell electromotility and otoacoustic emissions. EAR & HEARING.

[9] Blinowska KJ, DPDC, Fernandez BR (1994). The application of wavelet transform and matching pursuit to the time-varying EEG signals. Intelligent Engineering Systems through Artificial Neural Networks. ASME Press.

[10] Bohne B CW (1982).

[11] Fornaro M, Martino M (2010). Tinnitus psychophar- macology: A comprehensive review of its pathomechanisms and management. Neuropsychiatr Dis Treat.

[12] Moller AR (1997). Similarities between chronic pain and tinnitus. Am J Otolaryngol.

[13] Kemp (1981). Otoacoustic Emissions: Concepts and Origins. Ciba Found Symp.

[14] Leontien I, de Kleine EF, Rolien H, Van Dijk, Pim (2011). Contralateral suppression of otoacoustic emissions in tinnitus patients. Otol Neurotol.

[15] Borka J, Ceranic DKP, Ewa Raglan, Linda M Luxon (1998). Tinnitus after head injury: evidence from otoacoustic emissions. Neurol Neurosurg Psychiatry.

[16] Sztuka A, Pospiech L, Gawron W, Dudek K (2010). DPOAE in estimation of the function of the cochlea in tinnitus patients with normal hearing. Auris Nasus Larynx.

[17] Gouveris H MJ, Mann W (2005). DPOAE-grams in patients with acute tonal tinnitus. Otolaryngol Head Neck Surg.

[18] Fernandes Lda C ST (2009). Tinnitus and normal hearing: a study on the transient otoacoustic emissions suppression. Braz J Otorhinolaryngol.

[19] Ami M AA, Awang MA, Liyab B, Saim L (2008). Relation of distortion product otoacoustic emission with tinnitus. larngoscope.

[20] Hesse G AR, Schaaf H, Laubert A (2008). DPOAE and lateral inhibition in chronic tinnitus. hno.

